# A protective mechanism of probiotic *Lactobacillus* against hepatic steatosis via reducing host intestinal fatty acid absorption

**DOI:** 10.1038/s12276-019-0293-4

**Published:** 2019-08-13

**Authors:** Hye Rim Jang, Hyun-Jun Park, Dongwon Kang, Hayung Chung, Myung Hee Nam, Yeonhee Lee, Jae-Hak Park, Hui-Young Lee

**Affiliations:** 1 Laboratory of Mitochondrial and Metabolic Diseases, Department of Health Sciences and Technology, GAIHST, Gachon University, Incheon, Korea; 2 Department of Medicine, Gachon University School of Medicine, Incheon, Korea; 3 Seoul Center, Korea Basic Science Institute, Seoul, Korea; 4 Culture Collection of Antimicrobial Resistant Microbes, Department of Horticulture, Biotechnology and Landscape Architecture, Seoul Women’s University, Seoul, Korea; 5 Korea Mouse Metabolic Phenotyping Center, Lee Gil Ya Cancer and Diabetes Institute, Gachon University, Incheon, Korea; 6 Department of Laboratory Animal Medicine, College of Veterinary Medicine, Seoul National University, Seoul, Korea

**Keywords:** Metabolic disorders, Fat metabolism

## Abstract

The gut microbiome has been known to contribute up to ~30% of the energy absorption of the host. Although various beneficial mechanisms of probiotics have been suggested for non-alcoholic fatty liver disease (NAFLD), whether and which probiotics impact the host’s intestinal energy absorption have not yet been quantitatively studied. Here, we suggest a novel mechanism of probiotics against NAFLD, in which *Lactobacillus rhamnosus* GG, the most common probiotic, shares intestinal fatty acids and prevents the development of diet-induced hepatic steatosis. By using quantitative methods (radioactive tracers and LC–MS) under both in vitro and in vivo conditions, we found that bacteria and hosts competed for fatty acid absorption in the intestine, resulting in decreased weight gain, body fat mass, and hepatic lipid accumulation without differences in calorie intake and excretion in mice fed the probiotic bacteria.

## Liver disease: Beneficial bacteria divert dietary fats

Intestinal bacteria that consume common fatty acids could help protect their hosts against non-alcoholic fatty liver disease (NAFLD). The accumulation of lipids over the course of NAFLD can produce serious inflammation and ultimately lead to liver failure. The gut microbiome plays a prominent role in metabolic health and South Korean researchers led by Hui-Young Lee of Gachon University, Incheon, and Jae-Hak Park of Seoul National University, Seoul, have identified a bacterial species that could help prevent NAFLD. They found that *Lactobacillus rhamnosus* GG can absorb oleic acid, a fatty acid commonly found in the human diet. Transplantation of these bacteria into high-fat diet-fed mice resulted in weight loss and reduced fat accumulation in the liver. These findings providing a new mechanistic insight into the impact of certain probiotic species on NAFLD.

## Introduction

Non-alcoholic fatty liver disease (NAFLD), which is characterized by fat accumulation in the liver without significant alcohol consumption, is the most common liver disease in the world^1^. With the increased prevalence of obesity, the number of patients with NAFLD has rapidly increased over the past 20 years, with an estimated prevalence of ~25–30%^2^. The prevalence of NAFLD in patients with other metabolic diseases, such as obesity, type 2 diabetes, and hyperlipidemia, is greatly increased^3^. NAFLD includes a broad range of liver disease from simple steatosis to inflammatory steatohepatitis. Although simple steatosis is the mildest form of NAFLD, it is important in the pathogenesis of nonalcoholic steatohepatitis, which is distinguished by the presence of hepatocyte injury (ballooning degeneration of hepatocytes), inflammation, and/or fibrosis^1,4^. Simple steatosis is characterized by the deposition of triglycerides (TGs) as lipid droplets in the cytoplasm of hepatocytes^1,5^. A key process of simple steatosis is an imbalance between fatty acid input (synthesis and uptake) and output (export and oxidation)^6,7^; thus, intestinal lipid absorption is important in the development of the initial progress of NAFLD. Excessive dietary lipid absorption causes fat accumulation in extraintestinal tissues, such as liver and adipose tissue, which contributes to the development of simple steatosis and obesity^8^. Therefore, reducing intestinal lipid absorption would be an etiological strategy for developing a drug against NAFLD and associated metabolic diseases.

Probiotics, live microorganisms, confer health benefits, such as modulation of the gut microbiota, and antiobesity effects on the host and may serve as a potential alternative therapy for disease prevention and treatment^9^. Currently, both clinical and basic research have revealed several distinct cellular and molecular mechanisms underlying the beneficial effects of probiotics, including blocking pathogenic bacterial effects, regulating immune responses, and modulating intestinal epithelial homeostasis^10^. Long-term administration of *Lactobacillus* species, the most widely used probiotics^11^, has been reported to protect mice from NAFLD induced by a high-fat diet (HFD) and improve gut permeability, inflammation, and modulation of gut flora in a diet-induced obesity model^12–14^. However, existing studies on NAFLD focused on mainly inflammatory defense despite imperceptible inflammatory phenotypes in HFD-fed rodent models^15,16^ and did not quantitatively study whether probiotic *Lactobacillus* had any effect on the early progression of steatosis via the regulation of intestinal lipid absorption in vivo. Furthermore, despite previous studies showing that the gut microbiome accounts for 30% of the energy absorption of the host^17,18^, there is little research on which intestinal microorganisms can affect the host’s energy absorption. Oleic acid (OA), one of the major fatty acids in the diet^19^, has been suggested to incorporate into the cell membrane of *Lactobacillus* in vitro^20^, where it is further converted to cyclopropane fatty acids^21,22^, or to increase their survivability against acidic environmental conditions^23,24^. OA has also been commonly used as a major supplement for the growth media of *Lactobacillus* species (e.g., MRS broth, Difco, Detroit, MI). These observations lead us to hypothesize that *Lactobacillus* reduces intestinal lipid absorption, thereby protecting against diet-induced steatosis in vivo. Thus, we quantitatively examined whether *L. rhamnosus* GG consumes exogenous OA in a HFD-fed mouse model using radioactive tracers both in vitro and in vivo.

## Methods

### Mass spectrometry-based measurement of fatty acids in tissues and bacterial cultures

Long-chain acyl-CoAs (LCACoAs) and diacylglycerides were extracted from snap frozen liver tissues of both mice fed regular chow and mice fed a 60% HFD for one week. LCACoAs were purified using a solid phase extraction method described previously; OPC columns (Applied Biosystems, Foster City, CA) were used for the solid phase extraction^25^. Diacylglycerides were extracted using the Folch method^26^. Liver LCACoA and diacylglyceride contents were measured using a bench-top tandem mass spectrometer, 4000 Q TRAP (Applied Biosystems, Foster City, CA), as previously described^25^. The consumption of fatty acids by *L. rhamnosus* GG was measured by an ultra-performance liquid chromatography/quadrupole time-of-flight mass spectrometry (UPLC-Q-TOF/MS, Synapt G2Si, Waters, USA)-based metabolite profiling. One milliliter of cultured broth was centrifuged (10 min, 4000×*g*) every 3 h for 12 h, and metabolite profiles of the supernatant were analyzed with an UPLC Q/TOF–MS system in ESI (-) mode. Mass data, including retention time (RT), *m*/*z*, and ion intensities, were extracted using Progenesis QI software packages (Waters), and the peak of each fatty acid was selected on the basis of RT and accurate mass.

### Fatty acid and glucose consumption during bacterial cultivation

Fatty acid consumption of *Lactobacillus* strains, *L. rhamnosus* GG (ATCC 53103), *L. acidophilus* (ATCC 4356), and *L. gasseri* (ATCC 33323) in bacterial growth medium was evaluated quantitatively using radioactive tracers, [^14^C]-OA and [^14^C]-palmitic acid (PA) (PerkinElmer, Waltham, MA). The glucose concentration in each bacterial medium was measured using a GM9 glucose analyzer (Analox Instruments, London, UK). Overnight cultures of *Lactobacillus* were diluted 100-fold (v/v) and subcultured three times to achieve viability, as described previously^27^. [^14^C]-OA (1 µci) was added to 10 ml of MRS growth medium at 20 g/l, and three *Lactobacillus* strains (1x10^8^ cfu of each strain) were cultured for 6 h with shaking (37 °C at 220 rpm). For *L. rhamnosus GG*, 1 μci of [^14^C]-OA and [^14^C]-PA were further tested. One milliliter of cultured broth was aliquoted every 3 h and centrifuged (10 min, 4000×*g*), and the supernatant was collected. The pellet was resuspended and washed three times in MRS broth (10 min, 4000×*g*). ^14^C radioactivity was measured in both the supernatant and pellet of each aliquoted sample using a β-counter (Beckman scintillation counter, PerkinElmer, Waltham, MA).

### Fatty acid accumulation in cultured intestinal cells

To test whether the fatty acid consumption capacity of *L. rhamnosus* GG affects cellular fat accumulation in vitro, C2BBe1 cells (cloned from intestinal CaCo-2 cells, ATCC CRL-2102) were cocultured with *L. rhamnosus* GG using a 0.4 µm pore insert to exclude direct interactions between C2BBe1 cells and surface particles of *L. rhamnosus* GG, with minor modifications, as previously described^28,29^. Briefly, C2BBe1 cells were cultured at 37 °C under 5% CO_2_ in DMEM supplemented with 10% FBS, 10 μg/ml streptomycin, 10 U/ml penicillin (Welgene, Daegu, Korea), and 0.01 mg/ml human transferrin (Sigma-Aldrich, St Louis, MO). For experimentation, the cells were plated at a density of 2 × 10^5^ cells per well into six-well plates and were grown for 7 days postconfluence in culture medium. The experimental medium was prepared as follows: 100 μl of *L. rhamnosus* GG culture, grown to a concentration of 2 × 10^8^ cfu/ml in MRS broth, was added to 10 ml of DMEM containing 500 μmol/l OA (Sigma-Aldrich, St. Louis, MO), and the pH was adjusted to 7.4. Approximately 2 × 10^8^ cfu/ml *L. rhamnosus* GG was seeded on a Transwell membrane (SPL, Pochon, Korea) and inserted into a six-well culture plate containing C2BBe1 cells. As a control group, formalin-killed *L. rhamnosus* GG were prepared by immersion into 10% formalin for 1 h, followed by washing five times with PBS, and then resuspension into culture medium at a concentration of 1 × 10^9^ cfu/ml. To confirm bacterial death, formalin-killed bacteria were cultured in MRS broth for 24 h. After 6 h, C2BBe1 cells cocultured with *L. rhamnosus* GG under OA-treated conditions were collected, and TG extraction and quantification was performed according to the manufacturer’s protocol (Cayman Chemical, Ann Arbor, MI).

### Animals

Two animal studies were performed regarding HFD feeding. The long-term HFD study was performed for 9 weeks to evaluate the chronic effects of *L. rhamnosus* treatment on hepatic lipid accumulation and obesity. A short-term HFD study was performed for 1 week to determine whether intestinal lipid absorption is an underlying mechanism without differences in body weight or inflammatory status. To match ages on the experimental day, 5-week-old and 13-week-old male C57BL/6J mice were purchased from Jackson Laboratory (Bar Harbor, ME) and used for the long-term and short-term studies, respectively. In each study, HFD-fed mice were divided into two groups: a live *L. rhamnosus* GG-treated group (LGG, 1 × 10^9^ cfu/mouse/day) and a formalin-killed *L. rhamnosus* GG-treated group (fLGG, 1 × 10^9^ cfu/mouse/day). Regular chow diet (RCD)-fed mice were given saline instead of the bacteria. Each *L. rhamnosus* GG and saline treatment was administered daily to mice by oral gavage during the study period. Mice were individually housed in a specific pathogen-free facility under controlled temperature (22 ± 1 °C), humidity (55 ± 10%), and lighting (12-h light/dark) with free access to water and fed ad libitum with the HFD (45%, D12451, Research Diets, New Brunswick, NJ, for the long-term study; 60%, D12492, Research Diets, for short-term study) or regular chow diet (5053, Labdiet, St. Louis, MO). Body weight and food intake were monitored weekly. Body fat composition was measured by ^1^H-NMR (Bruker Optics, Billerica, MA). All animal experimental procedures were approved by the Institutional Animal Care and Use Committee at Gachon University and Seoul National University.

### Oil red o and Hematoxylin and eosin staining

In vitro, C2BBe1 cells were cocultured with *L. rhamnosus* GG under OA-treated conditions for 6 h, and then Oil Red O (Sigma-Aldrich, St. Louis, MO) staining was performed according to the manufacturer’s protocol. Oil Red O deposits were observed via light microscopy in the phase contrast view. For in vivo samples, paraffin sections of livers were stained with hematoxylin and eosin as described previously^27^. Cryosections of livers were stained with Oil Red O to visualize lipid droplets as described previously^30^. For individual scores for histopathological evaluation of hepatic steatosis, slices of the liver tissue were scored according to the criteria described previously^31^. Briefly, steatosis (0–3), lobular inflammation (0–2), hepatocellular ballooning (0–2), and fibrosis (0–4) were separately scored, and the NAFLD activity score (NAS) was expressed as the sum of each score.

### Quantitative measurement of intestinal lipid and glucose absorption in vivo

In the short-term HFD study, the LGG and fLGG groups were fasted overnight following 1 week of HFD feeding. Ten microcuries of [^14^C]-OA and 100 μci of [^3^H]-glucose (PerkinElmer, Waltham, MA) were dried and resuspended in 5 ml of MRS broth. After collection of fasting blood samples (0 min), 500 µl of the mixture (10 µci of [^3^H]-glucose and 1 µci of [^14^C]-OA) was orally administered to mice of each group. Intestinal fatty acid and glucose absorption were determined by measuring the plasma occurrence of [^14^C]-OA and [^3^H]-glucose radioactivity at 30, 60, 90, and 120 min. Tracer assays were performed after extracting the organic phase for [^14^C]-OA and after deproteinizing using barium hydroxide and zinc sulfate for [^3^H]-glucose, as previously described^7,32^.

### Basal plasma parameters

Blood samples collected by cardiac puncture from overnight-fasted mice were centrifuged for 20 min at 3000×*g* and stored at −20 °C. Total cholesterol, TG, high-density lipoprotein (HDL) cholesterol, low-density lipoprotein (LDL) cholesterol, aspartate transaminase (AST), and alanine transaminase (ALT) were measured in plasma using a Cobas c111 analyzer (Roche Diagnostics, Rotkreuz, Switzerland). Plasma nonesterified fatty acid (NEFA) content was measured using a colorimetric assay kit (Wako, Osaka, Japan).

### Quantitative RT-PCR

Total RNA was extracted from C2BBe1 cells and snap-frozen small intestine jejunums, livers, brown adipose tissues, and gastrocnemius skeletal muscle of overnight-fasted animals using TRIzol Reagent (Thermo Fishers Scientific, Waltham, MA). RNA was quantified by 260/280 wavelength measurement using a NanoDrop 2000C spectrophotometer (Thermo Fisher Scientific, Waltham, MA). Pure RNA was reverse transcribed using a TOPscript^TM^ RT DryMIX kit according to the manufacturer’s protocol (Enzynomics, Daejeon, Korea). Real-time PCR was performed on an applied Biosystems 7300 Real-time PCR system (Thermo Fishers Scientific, Waltham, MA). Primer sequences are listed in Supplementary Table 3.

### Fecal microbiota composition and fecal calorie excretion analyses

In the short-term HFD study, fecal samples from three groups were collected for 24 h following 1 week of HFD feeding. Samples from five mice in each group were combined, and the fecal microbiota composition was analyzed by Illumina sequencing performed by GenomicWorks (GenomicWorks, Daejeon, Korea) as described previously^33–36^. Briefly, PCR amplification was performed with extracted DNA using primers targeting the V3–V4 regions of the 16S rRNA gene. The amplified products were purified with a QIAquick PCR purification kit (Qiagen, Valencia, CA) and assessed on a Bioanalyzer 2100 (Agilent, Palo Alto, CA) using a DNA 7500 chip. Sequencing was carried out with an Illumina MiSeq Sequencing System according to the manufacturer’s instructions (Illumina, San Diego, CA). To measure fecal exergy excretion, feces were collected during the last 48 h, and calorie content in dried feces was analyzed by bomb calorimetry using a Parr 6400 Calorimeter (Parr, Moline, IL).

### Statistics

All results are expressed as the mean ± SEM. The significance of the differences in mean values among two groups was evaluated by two-tailed unpaired Student's *t*-tests. More than two groups were evaluated by one-way or two-way ANOVA (in the case of two independent variables) followed by post hoc analysis (Bonferroni, GraphPad Prism 5.0). *P*-values<0.05 were considered significant.

## Results

### OA is the most abundant fatty acids in the liver of HFD-fed mice

**Fig. 1:**
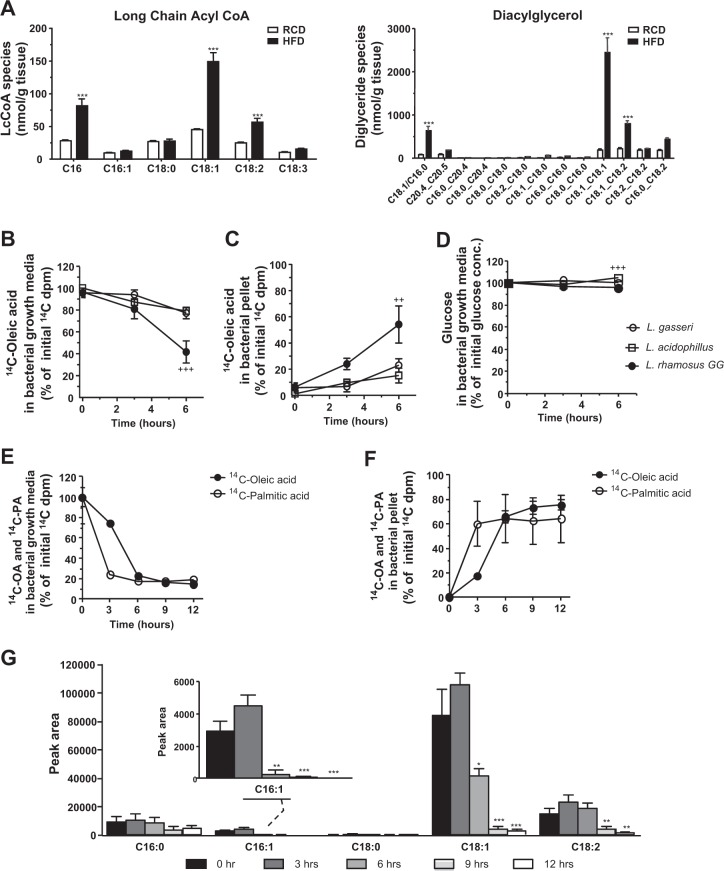
*Lactobacillus rhamnosus* GG consumes exogenous fatty acids. **a** Diet-dependent hepatic long-chain acyl CoA (left panel) and diacylglycerol (right panel) (*n* = 6 per group). [^14^C]-OA radioactivity measured in **b** bacterial growth media and in **c** bacterial pellets. **d** Percentage of glucose concentration measured in bacterial growth media. [^14^C]-OA and [^14^C]-PA radioactivity measured in **e** bacterial growth media and in **f** bacterial pellets. **g** Relative peak area of fatty acids detected in the growth media after 0, 3, 6, 9, and 12 h of incubation of *L. rhamnosus* GG (*n* = 6 per group). Data are expressed as the mean ± SEM from three independent experiments. ^++^*P* < 0.01 and ^+++^*P* < 0.001 for the difference between *L. rhamnosus GG* and others by two-way ANOVA and **P < 0.01 and ***P < 0.001 by one-way ANOVA with post hoc analysis (Bonferroni)

OA is a common dietary unsaturated fatty acid in human diets and is present in many rodent HFDs (i.e., D12492) used in metabolic studies^37^. OA accounts for ~34% of the composition of fatty acids in the HFD (Supplementary Table 1). Hepatic fatty acid composition reflected the diet. Specifically, OA (C18:1) was the most abundant long chain fatty acid in the HFD-fed mouse livers and was dramatically increased during HFD feeding in the liver (Fig. 1a, left). Furthermore, we also showed that OA was the most abundant fatty acid among the diacylglycerol species (Fig. 1a, right), which are known to play causative roles in insulin resistance, a major culprit of metabolic diseases^7,38,39^. PA, another key player in metabolic diseases^40,41^, occupied the highest percentage of saturated fatty acids in both the diet and liver (Fig. 1a, left). These results suggest that the dietary fatty acids ingested reflect fatty acid composition in the liver.

### *Lactobacillus* strains consume exogenous OA in bacterial growth media

Since it has been suggested that *Lactobacillus* strains use an exogenous OA source that is converted to cyclopropane fatty acid to increase their survival in acidic conditions in vitro^23^, we quantitatively measured whether *Lactobacillus* strains consume OA under normal growth conditions by using an [^14^C]-OA tracer. We cultured three *Lactobacillus* strains, namely, *L. gasseri*, *L. acidophilus*, and *L. rhamnosus* GG, in MRS broth with isotope-labeled [^14^C]-OA for 6 h and measured the radioactivity of [^14^C]-OA in bacterial growth media and bacterial pellets. During 6 h of growth, [^14^C]-OA radioactivity in the bacterial growth medium was markedly decreased by ~60% compared to the initial [^14^C]-OA activity by *L. rhamnosus* GG strains (Fig. 1b). The decreased radioactivity in the growth medium was associated with increased radioactivity in bacterial pellets (Fig. 1c), which indicates the incorporation of exogenous OA into the bacteria. However, the other *Lactobacillus* species, *L. gasseri* and *L. acidophilus*, showed only slight changes in [^14^C]-OA activity, accounting for ~20% decreases in the bacterial growth medium and ~20% increases in bacterial pellets during the 6 h of growth (Fig. 1b, c). The glucose concentration of the medium was similarly stable during growth of all three *Lactobacillus* strains, although *L. rhamnosus* GG showed slight decreases at 6 h (Fig. 1d). These results quantitatively indicate that *L. rhamnosus* GG consumes exogenous OA to a greater extent than other *Lactobacillus* strains in growth media.

### *Lactobacillus rhamnosus* GG consumes fatty acids during cultivation

To further test whether *L. rhamnosus* GG consumes specific fatty acid substrates, we cultured *L. rhamnosus* GG in MRS broth with isotope-labeled [^14^C]-OA and [^14^C]-PA for 12 h and measured the radioactivity in bacterial growth media and bacterial pellets. In bacterial growth medium, [^14^C]-PA radioactivity was decreased by ~70% at 3 h, and [^14^C]-OA radioactivity was decreased by ~70% at 6 h by *L. rhamnosus* GG (Fig. 1e) compared to the initial radioactivity. Each decrease in radioactivity in growth media was associated with increased radioactivity in bacterial pellets (Fig. 1f). Consistent with the radioactive tracer data, the fatty acid consumption of *L. rhamnosus* GG measured by UPLC-Q-TOF/MS showed similar results. While saturated fatty acid species, such as C16:0 and C18:0 in the media had a nonsignificant trend of decrease, fatty acid species containing double bonds (C18:1, C18:2, and C16:1) were significantly decreased over time in the bacterial growth medium (Fig. 1g). In particular, C16:1 and C18:1 showed earlier decreases than C18:2, even after 6 h of growth (Fig. 1g). These data quantitatively suggest that *L. rhamnosus* GG prefers fatty acids as its substrate during cultivation.

### *L. rhamnosus* GG reduces lipid-induced fat accumulation in intestinal C2BBe1 cells

**Fig. 2:**
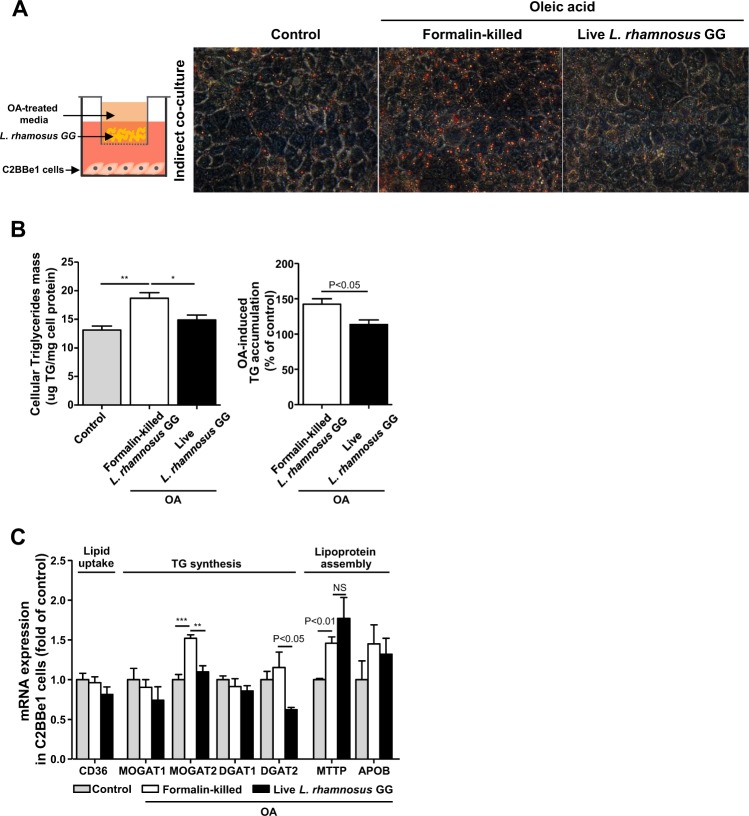
*Lactobacillus rhamnosus* GG reduces oleic acid-induced intestinal fat accumulation in vitro. **a** Oil Red O staining of OA-induced lipid accumulation in C2BBe1 cells cocultured indirectly with *L. rhamnosus* GG (original magnification ×40). **b** Cellular TG mass and OA-induced TG accumulation by the control. **c** mRNA levels of genes related to intestinal lipid metabolism in C2BBe1 cells. Data are expressed as the mean ± SEM from three independent experiments. *P < 0.05, **P < 0.01, and ***P < 0.001 by one-way ANOVA with post hoc analysis. Statistical comparisons obtained by Student’s *t*-test. NS, nonsignificant. Formalin-killed LGG, formalin-killed *L. rhamnosus* GG-treated C2BBe1 cells. Live LGG, live *L. rhamnosus* GG-treated C2BBe1 cells

Next, we investigated whether *L. rhamnosus* GG reduces lipid absorption at the cellular level using human intestinal C2BBe1 cells. To distinguish the effect of live bacteria on fatty acid consumption, we used both live *L. rhamnosus* GG and formalin-killed *L. rhamnosus* GG and cocultured them with C2BBe1 cells under OA-treated conditions for 6 h. Direct interaction of cells and bacteria was prevented by using an indirect coculture system (Fig. 2a). After 6 h of culture, OA-induced lipid accumulation, which was measured by both Oil Red O staining (Fig. 2a) and a TG kit (Fig. 2b), was significantly decreased by *L. rhamnosus* GG compared to that of formalin-killed *L. rhamnosus* GG. Accordingly, mRNA expression of monoacylglycerol acyltransferase (MOGAT) 2 and diacylglycerol acyltransferase (DGAT) 2, involved in TG synthesis, were significantly decreased in *L. rhamnosus* GG-treated C2BBe1 cells compared to that observed in formalin-killed *L. rhamnosus* GG-treated C2BBe1 cells (Fig. 2c). There were no differences in the expression of fatty acid uptake genes, such as cluster of differentiation 36 (CD36), and lipoprotein assembly genes, such as microsomal TG transfer protein (MTTP) or apolipoprotein B (APOB) (Fig. 2c). These results indicate that *L. rhamnosus* GG reduces OA-induced lipid accumulation in intestinal cells via limiting the exogenous OA source.

### Long-term feeding of *L. rhamnosus* GG reduces the gain of fat mass and hepatic lipid accumulation in HFD mice

**Fig. 3:**
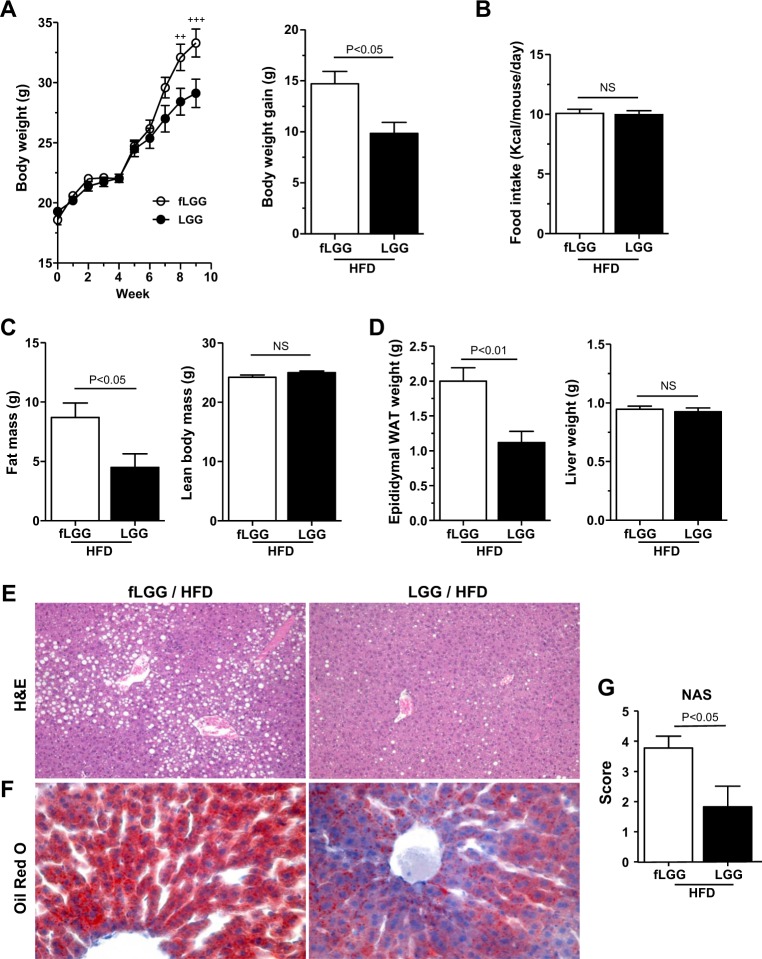
In vivo, body weight gain and fat mass are decreased in the LGG group during long-term HFD study. **a** Left panel: body weight change curve during experimental periods; right panel: body weight gain. **b** Food intake. **c** Body composition. The left panel is the fat mass, and the right panel is the lean body mass. **d** Tissue weight. The left panel is the epididymal white adipose tissue (WAT) weight, and the right panel is the liver tissue weight. **e** Hematoxylin and eosin staining and **f** Oil Red O staining of liver sections (original magnification ×20). **g** NAFLD activity score (NAS). Data are expressed as the mean ± SEM (*n* = 5–6 per group). ^++^*P* < 0.01 and ^+++^*P* < 0.001 by two-way ANOVA with post hoc analysis. Statistical analysis performed by Student’s *t*-test. NS, nonsignificant

Next, we assessed the effects of *L. rhamnosus* GG on the development of the level of diet-induced hepatic steatosis in vivo using a HFD-fed mouse model. During 9 weeks of HFD feeding, body weight began to diverge after 6 weeks and was significantly lower in the LGG group than in the fLGG group after 8 weeks of HFD feeding (Fig. 3a, left). After 9 weeks of HFD feeding, the LGG group exhibited an ~33% decrease in body weight gain compared to that of the fLGG group (Fig. 3a, right). Food intake during the experimental period was identical between the LGG and fLGG groups (Fig. 3b). The difference in body weight between the LGG and fLGG groups was ~5 g, which was mostly accounted for by decreased fat mass measured by ^1^H-NMR (Fig. 3c, left), as no difference in lean body mass was observed (Fig. 3c, right). Accordingly, epididymal white adipose tissue (WAT) weight was significantly decreased by ~50% in the LGG group compared to that in the fLGG group (Fig. 3d, left). Hepatic lipid accumulation revealed by Oil Red O staining (Fig. 3f) and NAS (Fig. 3g) was decreased in the LGG group compared to that in the fLGG group, mainly at the area of the central vein (Fig. 3e). We did not find any noticeable level of fibrosis or inflammatory foci in either the LGG or fLGG group (Fig. 3e). The liver weight between the LGG and fLGG groups was identical (Fig. 3d, right). These results indicate that *L. rhamnosus* GG protects against diet-induced hepatic lipid accumulation in the early stages of NAFLD.

### Short-term feeding of *L. rhamnosus* GG reduces intestinal lipid absorption without increasing fecal excretion

**Fig. 4:**
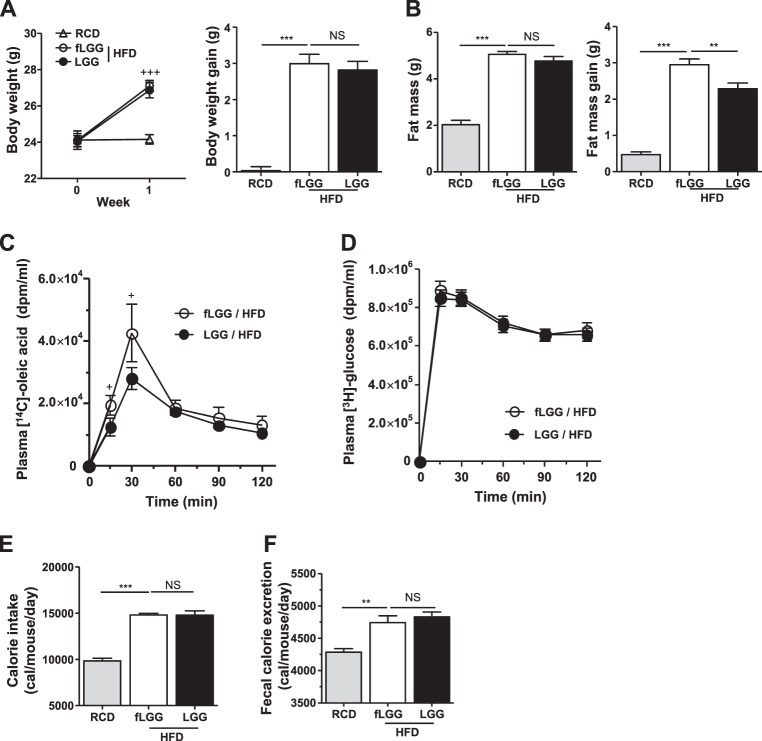
In vivo, *Lactobacillus rhamnosus* GG reduces fat mass gain and intestinal lipid absorption during a short-term HFD study. **a** Left panel: body weight change curve during experimental periods; right panel: body weight gain. **b** Left panel: fat mass; right panel: fat mass gain. **c** [^14^C]-OA radioactivity measured in plasma. **d** [^3^H]-glucose radioactivity measured in plasma. **e** Calorie intake. **f** Fecal calorie excretion. Data are expressed as the mean ± SEM (*n* = 5–7 per group). ^+^*P* < 0.05 and ^+++^*P* < 0.001 by two-way ANOVA and **P < 0.01 and ***P < 0.001 by one-way ANOVA with post hoc analysis. NS nonsignificant

Since long-term HFD feeding can influence the level of inflammation^42^, gut permeability^43^, composition of gut flora^44^, and body weight, all of which could have an effect on hepatic lipid accumulation, we chose an earlier period of HFD feeding, 1 week, to minimize these confounding factors and test whether *L. rhamnosus* GG inhibits intestinal OA absorption in vivo as was seen in vitro (Fig. 2). During 1 week of HFD feeding, body weight was similarly increased in both the fLGG and LGG groups (Fig. 4a); however, the gain of fat mass was slight but significantly reduced in the LGG group compared to that in the fLGG group (Fig. 4b). A [^14^C]-OA isotope was used to quantitatively measure the amount of lipid absorption from the intestinal rumen to the blood stream. Plasma [^14^C]-OA activity was significantly decreased in the LGG group compared to that in the fLGG group (Fig. 4c), while there were no differences in plasma [^3^H]-glucose (Fig. 4d), indicating no effect on glucose absorption. Both daily calorie intake (Fig. 4e) and fecal calorie excretion (Fig. 4f) were identical between the fLGG and LGG groups. Lean body mass, liver tissue weight, and circulation parameters, such as cholesterol, TG, NEFA, LDL, HDL, AST, and ALT levels, were identical between the fLGG and LGG groups (Supplementary Table 2). These results indicate that *L. rhamnosus* GG reduces intestinal OA absorption in the host by consuming the fatty acid in the intestinal rumen but not excreting it into feces.

### *L. rhamnosus* GG reduces the mRNA expression of genes related to lipid metabolism

**Fig. 5:**
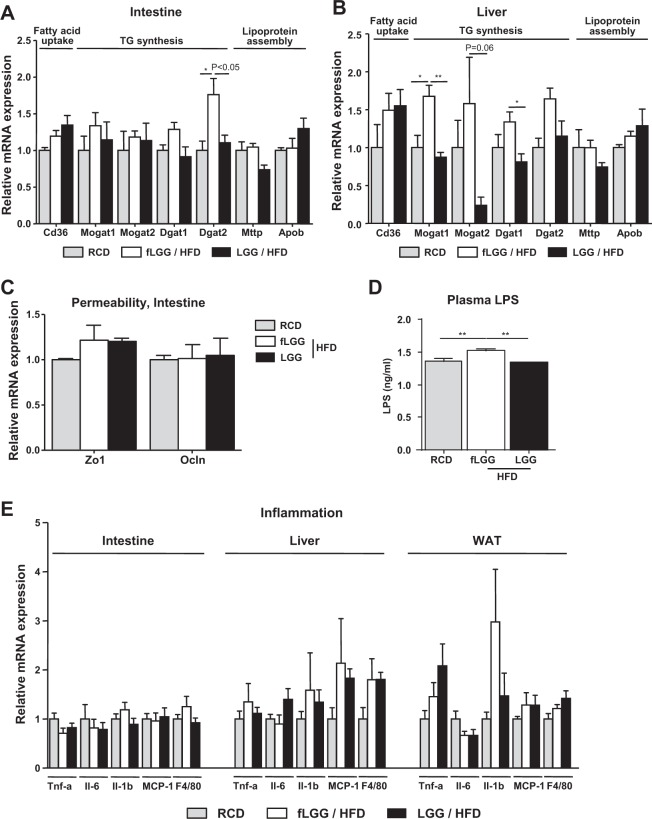
The effect of *L. rhamnosus* GG on lipid metabolism, gut permeability, and inflammation during short-term HFD feeding. mRNA expression of genes related to fatty acid uptake, TG synthesis and lipoprotein assembly in the **a** intestine and **b** liver. **c** mRNA expression of genes related to gut permeability. **d** Plasma LPS concentration. **e** mRNA expression of genes related to inflammation in the intestine, liver, and epididymal WAT. Data are expressed as the mean ± SEM (*n* = 5–6 per group). *P < 0.05 and **P < 0.01 by one-way ANOVA with post hoc analysis. Statistical analysis performed by Student’s *t*-test

In agreement with the gene expression data obtained from intestinal cells (Fig. 2c), the mRNA expression of lipid synthesis genes, *Dgat1* and *Dgat2*, after 1 week of HFD feeding was decreased in the intestines of the LGG group compared to that in the intestines of the fLGG group (Fig. 5a). There were similar changes in the liver, as *Mogat1*, *Mogat2*, *Dgat1*, and *Dgat2* gene expression was decreased in the LGG group (Fig. 5b). However, the mRNA expression of fatty acid uptake genes, such as *Cd36*, and lipoprotein assembly genes, such as *Apob* and *Mttp*, was not different among the groups in both the intestine and liver (Fig. 5a, b). Consistent with the intestine and liver, brown adipose tissue showed a similar mRNA expression pattern; TG synthesis-related gene expression was decreased in the LGG group compared to that in the fLGG group, but fatty acid uptake gene expression was not different among groups in brown adipose tissue (Fig. S1A). There were no differences in the expression of both fatty acid uptake and TG synthesis genes in gastrocnemius skeletal muscle (Fig. S1B). In the intestine, we further analyzed the expression of genes related to gut permeability^45^, namely, zonula occludens 1 (*Zo1*), tight junctions of intestinal cells, namely, occludin (*Ocln*), and inflammation, such as tumor necrosis factor-α (*Tnf-α*), interleukin (*Il*)*-6*, *Il-1β*, monocyte chemoattractant protein-1 (*MCP-1*), and F4/80, but none of these were different between the groups (Fig. 5c and e). Plasma LPS content was slightly reduced in the LGG group compared to that in the fLGG group (Fig. 5d). The mRNA expression of inflammatory markers was not changed in other tissues, including liver and WAT (Fig. 5e). These results indicate that *L. rhamnosus* GG affects intestinal lipid absorption of a host and the regulation of genes involved in TG synthesis but does not affect the expression levels of genes involved in gut permeability and inflammation during short-term HFD feeding.

### Diversity and population of the gut microbiota are altered by diet but exhibit reduced changes in the presence of *L. rhamnosus* GG treatment

**Fig. 6:**
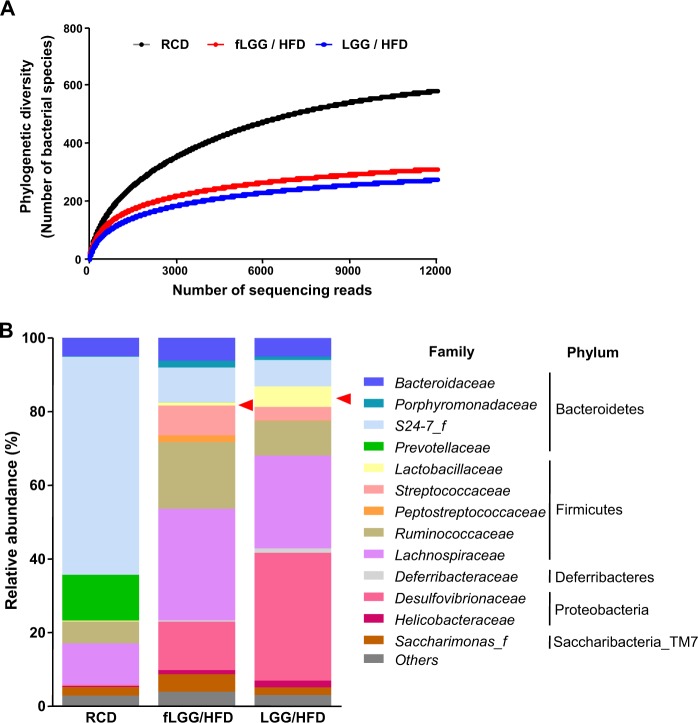
The effect of *L. rhamnosus* GG on the gut microbiota during short-term HFD feeding. **a** Rarefaction curves plotted for phylogenetic distance between the microbiota of the RCD, fLGG, and LGG groups. Phylogenetic distance was calculated at a rarefaction depth of 12,000 sequences/sample. **b** Comparison of family-level proportional abundance in the feces of the RCD, fLGG, and LGG groups (pooling, *n* = 3 per group). The red arrow indicates *Lactobacillaceae*

Since it is possible that altered gut microbiota due to probiotic administration could affect intestinal lipid absorption^46^, we investigated whether *L. rhamnosus* GG affected the phylogenetic richness of the gut microbiota during short-term HFD feeding. We analyzed α-diversity, as assessed by rarefaction and phylogenetic diversity, in each feces sample of three groups. Consistent with previously reported data^44^, we observed that the phylogenetic diversity was changed by diet and that the fecal microbiota of the HFD-fed groups (the fLGG and LGG groups) had lower phylogenetic diversity than that of the RCD group (Fig. 6a). However, the fecal microbiota of the fLGG and LGG groups was not different in phylogenetic diversity, which indicates that *L. rhamnosus* GG did not affect phylogenetic diversity in the fecal microbiota during short-term HFD feeding (Fig. 6a). Furthermore, we analyzed fecal microbiota composition and intestinal colonization of *L. rhamnosus* GG using 16S rRNA sequencing of feces after 1 week of HFD or RCD feeding. Similar to previous reports^46,47^, we found that the proportions of both Firmicutes and Proteobacteria were increased while the proportion of Bacteroidetes decreased in the HFD-fed groups (the fLGG and LGG groups) compared to those in the RCD group at the phylum level (Fig. 6b). At the family level, the proportion of *Lactobacillaceae* was markedly increased by ~5.6% in the LGG group, while it was almost undetectable in the RCD (~0.3%) and fLGG (~0.9%) groups (Fig. 6b). These data are consistent with our previous report that a single inoculation of *L. rhamnosus* GG colonized the intestine of mice and was detected in feces for up to 7 days^27^. Of the Proteobacteria, the LGG group showed an increase in the proportion of *Desulfovibrionaceae* (Fig. 6b), which has been previously reported to increase under HFD conditions;^48^ however, the effects on the beneficial results of the LGG group are not clear.

## Discussion

Intestinal microbiomes are composed of 10 times more microorganisms than the number of cells constituting the human body^49^. It is not surprising that the metabolism of the microbiome could affect the host's energy metabolism. Since the Jeffrey I. Gordon group published a paper on the discovery of obesity-related changes in the portion of gut bacteria belonging to Firmicutes and Bacteroidetes in human and animal^50–52^, many studies have confirmed that there is a notable difference in the intestinal compartment in obese mice compared to that in normal mice, with a significantly higher percentage of bacteria belonging to Firmicutes and a lower percentage to Bacteroidetes^47,53^. Furthermore, it is reported that normal mice receiving intestinal microflora from obese mice gain more weight than mice receiving intestinal microflora from lean mice even if they consume the same amount of calories^50^. In a study published in NEJM in 2014, researchers found that mice exposed to antibiotics in childhood become obese as adults, and normal mice become obese when grafted intestinal bacteria are transplanted into normal mice. These results indicate that intestinal microorganisms play an important role in energy accumulation and the development of obesity. Despite these studies over a decade, debate has remained regarding which specific bacteria and whether the increased bacteria have causality or consequence of obesity due to altered intestinal nutrients^17,53^. In particular, a decrease in Firmicutes has been recently reported in type 1 diabetes models, suggesting that a reduction in certain bacteria involved in fermentation could be a cause^54^. Furthermore, Bacteroidetes is known to be the major bacterium producing acetate (C2) and propionate (C3) in the intestine, which can induce glucose-stimulated insulin secretion and insulin resistance through activation of the parasympathetic nerve system and lipogenesis^55,56^. Firmicutes strains are known to produce mainly butyrate (C4), which improves insulin resistance by inhibiting HDAC^57,58^. *Lactobacillus*, which was used in this study, belongs to Firmicutes, and it has a characteristic of proliferating well when fatty acids are abundant and even using fatty acids when exposed to an acidic environment, such as small intestine's lumen^23^ (Fig. 1). Given the other positive features of *Lactobacillus* for other diseases when used as probiotics, increases in Firmicutes during a HFD, particularly the increase in *Lactobacillus*, are likely changes secondary to in intestinal lipid alterations rather than causes of obesity, even preventing obesity in the host by using its fatty acid-consuming abilities.

An open key question in probiotics is whether clinical application is able to improve metabolic research in humans. *L. rhamnosus* GG (LGG), ATCC 53103, was originally isolated from the feces of a healthy subject, and it was identified as a potential probiotic strain due to its resistance to acid and adhesion capacity to the intestinal epithelial layer^59^. Since then, the beneficial effects of this strain have been studied. However, in human intervention studies, its effects in obesity and related metabolic diseases were mild^60,61^, and applicable clinical symptoms are not exactly known yet. Since weight loss remains the mainstay of NAFLD treatment^62^, drugs reducing dietary lipid absorption could be a reasonable approach against hepatic steatosis. However, orlistat, a gastrointestinal lipase inhibitor, has been faced with side effects, such as oily stools and urgent bowel movements^63^. In this study, we clearly showed that, even under short-term periods, *L. rhamnosus* GG inhibits intestinal fatty acid absorption and decreases body fat accumulation without increasing fecal lipid excretion, which is a key determinant for the side effect of the orlistat. Mechanistically, it makes sense if gut bacteria consume the excessive intestinal lipids increased by the orlistat, steatorrhea could be improved. Thus, it could be a testable trial whether orlistat’s steatorrhea is decreased with combinational treatment of specific gut bacteria that consume fatty acids from among the probiotics, such as *L. rhamnosus* GG. More sophisticated lipid tracers that are currently under development may help to quantify energy uptake and identify the mechanism in humans.

Mechanistically, probiotic *Lactobacillus* strains have been proposed to benefit human health with several general mechanisms of action^11,64^. First, certain *Lactobacillus* can directly or indirectly influence the abundance or diversity of the commensal microbiota^65^. Second, certain *Lactobacillus* strains have the ability to enhance epithelial barrier function, such as via nuclear factor-κB (NF-kB) and mitogen-activated protein kinase (MAPK)-dependent pathways, which are related to the function of mucus or tight junctions in intestinal cells^65^. Third, most probiotic *Lactobacillus* strains can also modulate the host’s immune responses and exert local systemic effects specific to the strains^66^, which are mostly mediated by microbe-associated molecular patterns (MAMPs)^67^. Last, bacteria produce metabolites from intestinal carbohydrates, amino acids and lipid sources, such as short chain fatty acids (SCFAs), branched amino acids and conjugated linoleic acid^27,68^. Although many experiments with in vitro and in vivo animal models validate these mechanisms for probiotic strains in general and for *L. rhamnosus* GG in particular, most published data pay less attention to the characteristic outcome of *Lactobacillus* in fatty acid absorption. In our knowledge, only one previous study showed that *Lactobacillus reuteri* JBD301 reduces dietary fat absorption in the intestine and protects against diet-induced obesity under long-term HFD-feeding conditions^69^, which is consistent with our results in this study. However, their findings for fatty acid absorption were limited to in vitro evidence. Furthermore, it was difficult to distinguish whether host-bacterial fatty acid competition is primary or secondary to the anti-obesity effect of *Lactobacillus*, since *Lactobacillus* strains have been known to alter systemic inflammation, gut permeability, and the population of the gut microbiota under chronic HFD feeding conditions^12–14^. In this study, we attempted to dissociate these confounding factors and identify a new role for *L. rhamnosus* GG in the early progression of NAFLD by using acute HFD-fed and body weight-matched mice. Furthermore, we quantitatively demonstrated a novel mechanism by which *L. rhamnosus* GG reduces intestinal fatty acid absorption using tracer-labeled [^14^C]-OA in vivo, which was not associated with gut permeability or systemic inflammation under short-term HFD-feeding conditions. These results provide a novel mechanism by which *L. rhamnosus* GG consumes intestinal fatty acids and protects against the initial stage of NAFLD development earlier than changes in gut permeability or inflammation in vivo. Additionally, we showed that *L. rhamnosus* GG reduces body fat gain and protects against lipid-induced hepatic steatosis under long-term HFD-feeding conditions. Together, these data provide an additional and new function of *L. rhamnosus* GG in competing with the host for intestinal lipid uptake and suggest a therapeutic potency of the most well-known probiotics against diet-induced obesity, NAFLD, and related metabolic diseases.

## Supplementary information

Supplementary Figure Legends & Tables.

Supplementary Figure 1.
