# Supplementary material for: A protective mechanism of probiotic Lactobacillus against hepatic steatosis via reducing host intestinal fatty acid absorption
Source: Exp Mol Med. 2019 Aug 13;51(8):95. doi: 10.1038/s12276-019-0293-4 (PMC6802638; doi:10.1038/s12276-019-0293-4)
Supplement: Supplementary file 1 — Supplementary Figure Legends & Tables. [file 12276_2019_293_MOESM1_ESM.docx]

**Supplementary information**

**Supplementary Figure legends**

**Supplementary figure 1. The effect of *Lactobacillus rhamnosus* GG on lipid metabolism in brown adipose tissue and skeletal muscle during short-term HFD feeding.**

mRNA expression of genes related to fatty acid uptake, TG synthesis and fatty acid oxidation in (A) brown adipose tissue and (B) gastrocnemius skeletal muscle. Data are expressed as mean ± SEM (n = 5 - 6 per group). *P < 0.05 by One-Way ANOVA with post-hoc analysis. Statistical number by Student’s t-test.

***Supplementary table 1. The fatty acid composition of both 45% and 60% high-fat diet***

| Fatty acids | 45% HFD | | 60% HFD | |
| --- | --- | --- | --- | --- |
|  | (g) | (%) | (g) | (%) |
| C10, Capric | 0.1 | 0.05 | 0.1 | 0.04 |
| C12, Lauric | 0.2 | 0.1 | 0.2 | 0.08 |
| C14, Myristic | 2.0 | 1.05 | 2.8 | 1.1 |
| C15 | 0.1 | 0.05 | 0.2 | 0.08 |
| C16, Palmitic | 36.9 | 19.32 | 49.9 | 19.65 |
| C16:1, Palmitoleic | 2.4 | 1.26 | 3.4 | 1.34 |
| C17 | 0.7 | 0.37 | 0.9 | 0.35 |
| C18, Stearic | 19.8 | 10.37 | 26.9 | 10.59 |
| C18:1, Oleic | 64.4 | 33.72 | 86.6 | 34.09 |
| C18:2, Linoleic, n-6 | 56.7 | 29.69 | 73.1 | 28.78 |
| C18:3, Linolenic, n-3 | 4.3 | 2.25 | 5.2 | 2.05 |
| C20:1 | 1.1 | 0.58 | 1.5 | 0.59 |
| C20:2 | 1.4 | 0.73 | 2.0 | 0.79 |
| C20:3, n-6 | 0.2 | 0.1 | 0.3 | 0.12 |
| C20:4, Arachidonic, n-6 | 0.5 | 0.26 | 0.7 | 0.28 |
| C22:5, Docosapentaenoic, n-3 | 0.2 | 0.1 | 0.2 | 0.08 |
| Total | 191 | 100 | 254 | 100 |

Both 45% and 60% high-fat diet purchased from the Research Diets was used for animal experiments. The fatty acid composition data was provided by the company (Research Diets, New Brunswick, NJ).

***Supplementary table 2. Metabolic parameters of animals***

|  | RCD | fLGG / HFD | LGG / HFD | P-value  (RCD vs fLGG/HFD) | P-value  (fLGG/HFD vs LGG/HFD) |
| --- | --- | --- | --- | --- | --- |
| Cholesterol (mg/dL) | 113.028 ± 12.65 | 149.106 ± 11.53 | 154.73 ± 11.46 | NS | NS |
| TG (mg/dL) | 64.92 ± 1.69 | 72.394 ± 10.66 | 67.146 ± 4.49 | NS | NS |
| NEFA (mmol/L) | 0.759 ± 0.13 | 0.705 ± 0.09 | 0.668 ± 0.07 | NS | NS |
| LDL (mg/dL) | 9.226 ± 2.35 | 16.581 ± 5.09 | 17.129 ± 2.52 | NS | NS |
| HDL (mg/dL) | 103.844 ± 11.54 | 130.706 ± 9.03 | 137.856 ± 9.53 | NS | NS |
| ALT (U/L) | 26.88 ± 3.98 | 21.914 ± 1.71 | 31.857 ± 5.66 | NS | NS |
| AST (U/L) | 90.04 ± 26.81 | 75.514 ± 8.76 | 112 ± 17.22 | NS | NS |
| Liver weight (g) | 0.96 ± 0.01 | 0.92 ± 0.01 | 0.89 ± 0.02 | NS | NS |
| Lean body mass (g) | 22.13 ± 0.39 | 22.06 ± 0.31 | 22.11 ± 0.33 | NS | NS |

Blood samples collected by cardiac puncture from overnight-fasted mice. Data are expressed as mean ± SEM (n = 5 - 7). There was no significant difference among groups by One-Way ANOVA with post-hoc analysis. NS, non-significant. TG, triglyceride. NEFA, non-esterified fatty acids. LDL, low-density lipoprotein. HDL, high-density lipoprotein. AST, aspartate transaminase. ALT, alanine transaminase.

***Supplementary table 3. The primer sequences used in RT-PCR analyses***

| Gene | Forward Primer Sequence (5’ ->3’) | Reverse Primer Sequence (5’ ->3’) |
| --- | --- | --- |
| Human CD36 | GGCTGTGACCGGAACTGTG | TTCTGTGCCTGTTTTAACCCAA |
| Human MOGAT1 | AAAGTGTGTCCTACATGGTAAGC | TGATCCTTCAGGGTTGTCAGTT |
| Human MOGAT2 | ACACTTGCTGTCCTACAGTTTG | GAGGAGCCAGAATCTTGTAAACA |
| Human DGAT1 | TATTGCGGCCAATGTCTTTGC | CACTGGAGTGATAGACTCAACCA |
| Human DGAT2 | AGCAGGTGATCTTCGAGGAG | CATGGGGCGAAACCAATGTA |
| Human MTTP | ACAAGCTCACGTACTCCACTG | TCCTCCATAGTAAGGCCACATC |
| Human APOB | TGCTCCACTCACTTTACCGTC | TAGCGTCCAGTGTGTACTGAC |
| Human GAPDH | GGAGCGAGATCCCTCCAAAAT | GGCTGTTGTCATACTTCTCATGG |
| Mouse Cd36 | ATTGGTCAAGCCAGCT | TGTAGGCTCATCCACTAC |
| Mouse Mogat1 | TTGTGCTTTGGGGTGCTATCA | CCACAGTGGGAACTCTCCA |
| Mouse Mogat2 | TGGGAGCGCAGGTTACAGA | CAGGTGGCATACAGGACAGA |
| Mouse Dgat1 | TCCGTCCAGGGTGGTAGTG | TGAACAAAGAATCTTGCAGACGA |
| Mouse Dgat2 | GCGCTACTTCCGAGACTACTT | GGGCCTTATGCCAGGAAACT |
| Mouse Mttp | CTCTTGGCAGTGCTTTTTCTCT | GAGCTTGTATAGCCGCTCATT |
| Mouse Apob | TTGGCAAACTGCATAGCATCC | TCAAATTGGGACTCTCCTTTAGC |
| Mouse Zo1 | GCCGCTAAGAGCACAGCAA | TCCCCACTCTGAAAATGAGGA |
| Mouse Ocln | TTGAAAGTCCACCTCCTTACAGA | CCGGATAAAAAGAGTACGCTGG |
| Mouse Tnf-α | CCAGACCCTCACACTCAGATC | CACTTGGTGGTTTGCTACGAC |
| Mouse Il-6 | GGCGGATCGGATGTTGTGAT | GGACCCCAGACAATCGGTTG |
| Mouse Il-1β | AGGCAGGCAGTATCACTCATTGT | GGAAGGTCCACGGGAAAGA |
| Mouse MCP-1 | GCAGTTAACGCCCCACTCA | CCAGCCTACTCATTGGGATCA |
| Mouse F4/80 | TCATCAGCCATGTGGGTACAG | CACAGCAGGAAGGTGGCTATG |
| Mouse Fatp1 | TGCTTTGGTTTCTGGGACTT | GCTCTAGCCGAACACGAATC |
| Mouse Pgc1-a | CGATGTGTCGCCTTCTTGCT | CGAGAGCGCATCCTTTGG |
| Mouse Cpt1b | GCACACCAGGCAGTAGCTTT | CAGGAGTTGATTCCAGACAGGTA |
| Mouse Acox1 | TAACTTCCTCACTCGAAGCCA | AGTTCCATGACCCATCTCTGTC |
| Mouse Mcad | GGT TTG GCT TTT GGA CAA TG | TGA CGT GTC CAA TCT ACC ACA |
| Mouse Gapdh | ACCACAGTCCATGCCATCAC | TCCACCACCCTGTTGCTGTA |
| Mouse Cyclo A | CAAGACTGAATGGCTGGATG | TGGTGATCTTCTTGCTGGTC |
