# Supplementary figures and images for: A protective mechanism of probiotic Lactobacillus against hepatic steatosis via reducing host intestinal fatty acid absorption
Source: Exp Mol Med. 2019 Aug 13;51(8):95. doi: 10.1038/s12276-019-0293-4 (PMC6802638; doi:10.1038/s12276-019-0293-4)

Supplementary figure 1.

A

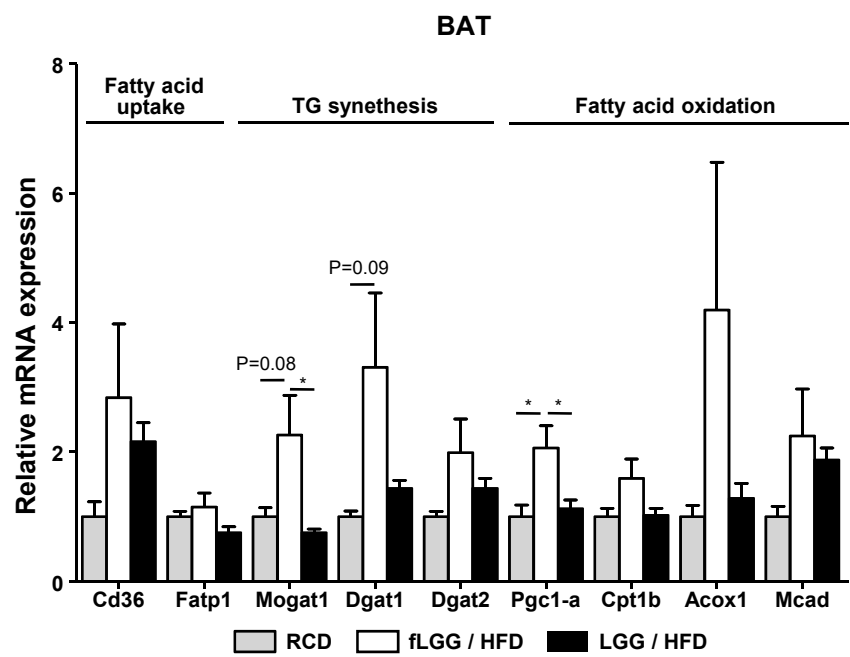

B

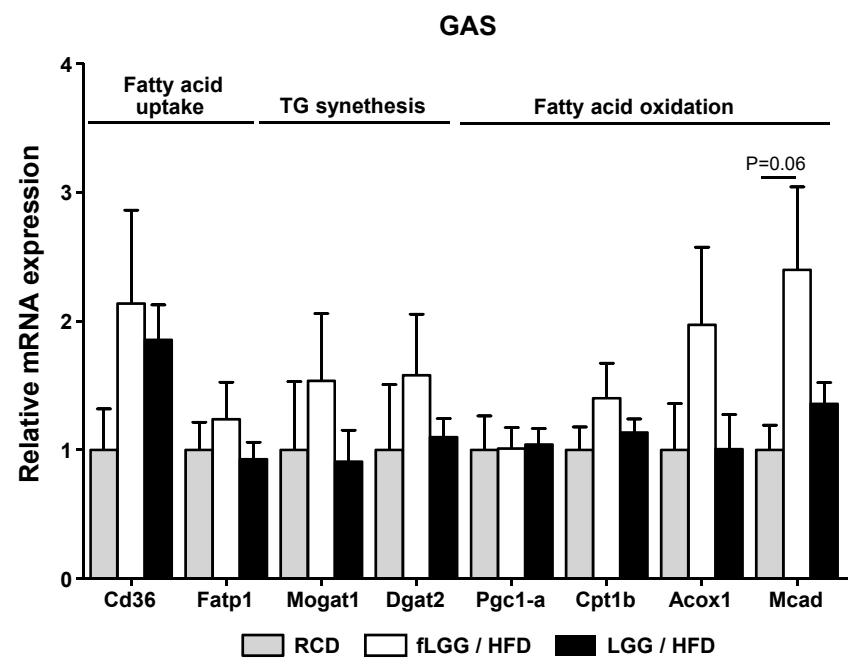

Supplement: Supplementary file 2 — Supplementary Figure 1. [file 12276_2019_293_MOESM2_ESM.pdf]
